# Bromide Substituted for Iodide of Potassium

**Published:** 1871-11

**Authors:** 


### Editorial Department.

### Bromide Substituted for Iodide of Potassium.

The following Circular handed us by Mr. W. H. Peabody, we consider worthy of the particular attention of the Profession. The present scarcity of Iodide has advanced the price of all its preparations, and Iodide of Potassium is now more than four times as expensive as the Bromide.

The temptation to unprincipled men to institute the one for the other is therefore great. When this medicine is prescribed and fails to produce its proper effects, we would recommend an immediate examination of the Drug.

PHILADELPHIA, November 3d, 1871.

DEAR SIR:—

We beg to advise you that we have been informed of several parcels of Bromide of Potassium being offered to some of our drug friends as Iodide of Potassium; the strip label of the former having been removed from the bottle, and one for Iodide of Potassium substituted.

As they are very similar in appearance we take the liberty of suggesting the propriety of a close examination of the labels as well as the article itself, should any be offered to you by irresponsible parties, particularly if at prices much below current rates.

For Iodide of Potassium we have the engraving of our Laboratory, and underneath it, but printed on the *same label*, the words Iodide of Potassium, &c.; while for Bromide of Potassium we have, up to this time, used a strip label above and separate from the engraving of the Laboratory. We are about changing our style for Bromide of Potassium, and expect in a short time to have the name of the article on the same label with the representation of the Factory, as for Iodide.

Should there be any doubt as to the purity of the article offered, or to its being the Iodide, the point can be readily determined by employing the Corrosive Sublimate test. This will be found on page 1299 of the U. S. Dispensatory, 12th edition.

Very respectfully,

Your friends,

POWERS & WEIGHTMAN.

